# Supplementary material for: Double-tap gene drive uses iterative genome targeting to help overcome resistance alleles
Source: Nat Commun. 2022 May 9;13:2595. doi: 10.1038/s41467-022-29868-3 (PMC9085836; doi:10.1038/s41467-022-29868-3)
Supplement: Supplementary file 1 — Supplementary Information [file 41467_2022_29868_MOESM1_ESM.pdf]

“Double-Tap” Gene Drive uses iterative genome targeting to help overcome resistance alleles

Alena L. Bishop[1],Víctor López Del Amo[1], Emily Okamoto[1], Zsolt Bodai[2], Alexis Komor[2], Valentino M. Gantz[1].*

[1] Division of Biological Sciences, Section of Cell and Developmental Biology, University of California San Diego, La Jolla, CA 92093, USA.

[2] Department of Chemistry and Biochemistry, University of California San Diego, La Jolla, CA 92093, USA.

*Correspondence: vgantz@ucsd.edu

Keywords: gene drive, resistance alleles, multiplexing, gRNAs, population modification, Drosophila, CRISPR.

Supplementary Figure 1

a

```
White NHEJ Sequences:

                                                                                                               # flies (# vials)
   WT:  ACAGGTTGGCCATTGAGCAGTCGCATCCCGGATGGCGATACTTGGATGCC                         CTGCGGCGATCGAAAGGCAAGGGCATTCAGCAGGGTCGTCTTTCCGGCAC

DT-tGD(y1,w2,y1b) [pVMG127]
        ACAGGTTGGCCATTGAGCAGTCGCATCCCGG···················                         ···CGGCGATCGAAAGGCAAGGGCATTCAGCAGGGTCGTCTTTCCGGCAC  -22             2  (1)
        ACAGGTTGGCCATTGAGCAGTCGCATCCCGGATGGCGATACTTGGATGC·                         ··GCGGCGATCGAAAGGCAAGGGCATTCAGCAGGGTCGTCTTTCCGGCAC  -3              4  (1)
        ACAGGTTGGCCATTGAGCAGTCGCATCCCGGATGGCGATACTTGGAT··· A                       CTGCGGCGATCGAAAGGCAAGGGCATTCAGCAGGGTCGTCTTTCCGGCAC  -3  +1          5  (2)
   w2b: ACAGGTTGGCCATTGAGCAGTCGCATCCCGGATGGCGATACTTGGATGCC                         ·TGCGGCGATCGAAAGGCAAGGGCATTCAGCAGGGTCGTCTTTCCGGCAC  -1             13  (7)
        ACAGGTTGGCCATTGAGCAGTCGCATCCCGGATGGCGATACTTGGATG·· GCC                     ··GCGGCGATCGAAAGGCAAGGGCATTCAGCAGGGTCGTCTTTCCGGCAC  -4  +3          1  (1)
        ACAGGTTGGCCATTGAGCAGTCGCATCCCGGATGGCGATACTTGG····· CGCCTTGGATA             CTGCGGCGATCGAAAGGCAAGGGCATTCAGCAGGGTCGTCTTTCCGGCAC  -5  +11         1  (1)
        ACAGGTTGGCCATTGAGCAGTCGCATCCCGGATGGCGATACTTGG····· CGATACTTGGCGATTTCGATA   CTGCGGCGATCGAAAGGCAAGGGCATTCAGCAGGGTCGTCTTTCCGGCAC  -5  +21         1  (1)

DT-tGD(y1,w2,w2b) [pVMG128]
        ACAGGTTGGCCATTGAGCAGTCGCATCCCGGATGGCGATACTTGGA···· ACT                     ··············································GCAC  -50 +3          1  (1)
        ACAGGTTGGCCATTGAGCAGTCGCATCCCGGATGGCGATACTTGGAT···                         ··········CGAAAGGCAAGGGCATTCAGCAGGGTCGTCTTTCCGGCAC  -13             1  (1)
        ACAGGTTGGCCATTGAGCAGTCGCATCCCGGATGGCGATACTTGG·····                         ······CGATCGAAAGGCAAGGGCATTCAGCAGGGTCGTCTTTCCGGCAC  -11             2  (2)
        ACAGGTTGGCCATTGAGCAGTCGCATCCCGGATGGCGATACTTGGATGC·                         ····GGCGATCGAAAGGCAAGGGCATTCAGCAGGGTCGTCTTTCCGGCAC  -5              5  (4)
        ACAGGTTGGCCATTGAGCAGTCGCATCCCGGATGGCGATACTTGGATG··                         ·TGCGGCGATCGAAAGGCAAGGGCATTCAGCAGGGTCGTCTTTCCGGCAC  -3              9  (1)
        ACAGGTTGGCCATTGAGCAGTCGCATCCCGGATGGCGATACTTGGATGC·                         ··GCGGCGATCGAAAGGCAAGGGCATTCAGCAGGGTCGTCTTTCCGGCAC  -3              2  (1)
        ACAGGTTGGCCATTGAGCAGTCGCATCCCGGATGGCGATACTTGGATG··                         CTGCGGCGATCGAAAGGCAAGGGCATTCAGCAGGGTCGTCTTTCCGGCAC  -2              4  (2)
        ACAGGTTGGCCATTGAGCAGTCGCATCCCGGATGGCGATACTTGGAT··· A                       CTGCGGCGATCGAAAGGCAAGGGCATTCAGCAGGGTCGTCTTTCCGGCAC  -3  +1          1  (1)
        ACAGGTTGGCCATTGAGCAGTCGCATCCCGGATGGCGATACTTGGAT··· C                       CTGCGGCGATCGAAAGGCAAGGGCATTCAGCAGGGTCGTCTTTCCGGCAC  -3  +1          4  (2)
        ACAGGTTGGCCATTGAGCAGTCGCATCCCGGATGGCGATACTTGGA···· GATA                    CTGCGGCGATCGAAAGGCAAGGGCATTCAGCAGGGTCGTCTTTCCGGCAC  -4  +4   (w+)   4  (1)
        ACAGGTTGGCCATTGAGCAGTCGCATCCCGGATGGCGATACTTGGATGCC C                       CTGCGGCGATCGAAAGGCAAGGGCATTCAGCAGGGTCGTCTTTCCGGCAC      +1          1  (1)
        ACAGGTTGGCCATTGAGCAGTCGCATCCCGGATGGCGATACTTGGATGC· GATA                    CTGCGGCGATCGAAAGGCAAGGGCATTCAGCAGGGTCGTCTTTCCGGCAC  -1  +4   (w+)   1  (1)
        ACAGGTTGGCCATTGAGCAGTCGCATCCCGGATGGCGATACT········ GCGGATGGATA             CTGCGGCGATCGAAAGGCAAGGGCATTCAGCAGGGTCGTCTTTCCGGCAC  -8  +11  (w+)   2  (1)
        ACAGGTTGGCCATTGAGCAGTCGCATCCCGGATGGCGATACTTGGAT··· CTTGCGGTACT             ·TGCGGCGATCGAAAGGCAAGGGCATTCAGCAGGGTCGTCTTTCCGGCAC  -4  +11         1  (1)

DT-tGD(y1,w2,y1b,w2b) [pVMG129]
        ACAGGTTGGCCATTGAGCAGTCGCATCCCGGATGGCGATACTTGG·····                         ···CGGCGATCGAAAGGCAAGGGCATTCAGCAGGGTCGTCTTTCCGGCAC  -8              6  (2)
        ACAGGTTGGCCATTGAGCAGTCGCATCCCGGATGGCGATACTTGGATGC·                         ····GGCGATCGAAAGGCAAGGGCATTCAGCAGGGTCGTCTTTCCGGCAC  -5              7  (4)
        ACAGGTTGGCCATTGAGCAGTCGCATCCCGGATGGCGATACTTGGATGC·                         ·TGCGGCGATCGAAAGGCAAGGGCATTCAGCAGGGTCGTCTTTCCGGCAC  -2              3  (1)
        ACAGGTTGGCCATTGAGCAGTCGCATCCCGGATGGCGATACTTGGAT··· A                       CTGCGGCGATCGAAAGGCAAGGGCATTCAGCAGGGTCGTCTTTCCGGCAC  -3  +1          3  (1)
        ACAGGTTGGCCATTGAGCAGTCGCATCCCGGATGGCGATACTTGGATGC· ATCGCA                  ·TGCGGCGATCGAAAGGCAAGGGCATTCAGCAGGGTCGTCTTTCCGGCAC  -2  +6          2  (1)
```

b

```
Yellow NHEJ Sequences:

                                                                                                               # flies (# vials)
   WT:  CCGCATTAAAGTGGATGAGTGTGGTCGGCTGTGGGTTTTGGACACTGGAA                         CCGTGGGCATCGGCAATACCACCACTAATCCGTGCCCCTATGCGGTAAAT

DT-tGD(y1,w2,y1b) [pVMG127]
        CCGCATTAAAGTGGATGAGTGTGGTCGGCTGTGGGTTTTGGACACTGG··                         ······GCATCGGCAATACCACCACTAATCCGTGCCCCTATGCGGTAAAT  -8              5  (1)
        CCGCATTAAAGTGGATGAGTGTGGTCGGCTGTGGGTTTTGGACAC·····                         ·CGTGGGCATCGGCAATACCACCACTAATCCGTGCCCCTATGCGGTAAAT  -6              6  (3)
        CCGCATTAAAGTGGATGAGTGTGGTCGGCTGTGGGTTTTGGACACTG···                         ·CGTGGGCATCGGCAATACCACCACTAATCCGTGCCCCTATGCGGTAAAT  -4              1  (1)
        CCGCATTAAAGTGGATGAGTGTGGTCGGCTGTGGGTTTTGGACAC····· A                       CCGTGGGCATCGGCAATACCACCACTAATCCGTGCCCCTATGCGGTAAAT  -5  +1          2  (1)
        CCGCATTAAAGTGGATGAGTGTGGTCGGCTGTGGGTTTTGGACACTGG··                         CCGTGGGCATCGGCAATACCACCACTAATCCGTGCCCCTATGCGGTAAAT  -2              3  (1)
        CCGCATTAAAGTGGATGAGTGTGGTCGGCTGTGGGTTTTGGACACTGGAA                         ··GTGGGCATCGGCAATACCACCACTAATCCGTGCCCCTATGCGGTAAAT  -2              1  (1)
        CCGCATTAAAGTGGATGAGTGTGGTCGGCTGTGGGTTTTGGACACTGG·· CAT                     ·CGTGGGCATCGGCAATACCACCACTAATCCGTGCCCCTATGCGGTAAAT  -3  +3   (y+)   1  (1)
        CCGCATTAAAGTGGATGAGTGTGGTCGGCTGTGGGTTTTGGACACTGGAA A                       CCGTGGGCATCGGCAATACCACCACTAATCCGTGCCCCTATGCGGTAAAT      +1          4  (2)
        CCGCATTAAAGTGGATGAGTGTGGTCGGCTGTGGGTTTTGGACACTGGA· CA                      CCGTGGGCATCGGCAATACCACCACTAATCCGTGCCCCTATGCGGTAAAT  -1  +2          2  (1)
        CCGCATTAAAGTGGATGAGTGTGGTCGGCTGTGGGTTTTGGACACTGGA· TG                      CCGTGGGCATCGGCAATACCACCACTAATCCGTGCCCCTATGCGGTAAAT  -1  +2          1  (1)
        CCGCATTAAAGTGGATGAGTGTGGTCGGCTGTGGGTTTTGGACACTGG·· GTTTGACA                ···TTGGCATCGGCAATACCACCACTAATCCGTGCCCCTATGCGGTAAAT  -5  +8          1  (1)
        CCGCATTAAAGTGGATGAGTGTGGTCGGCTGTGGGTTTTGGACACTGGA· CACTGGA                 ·CGTGGGCATCGGCAATACCACCACTAATCCGTGCCCCTATGCGGTAAAT  -2  +7          1  (1)

DT-tGD(y1,w2,w2b) [pVMG128]
        CCGCATTAAAGTGGATG·································                         CCGTGGGCATCGGCAATACCACCACTAATCCGTGCCCCTATGCGGTAAAT  -33             1  (1)
        CCGCATTAAAGTGGATGAGTGTGGTCGG······················ ATGC                    CCGTGGGCATCGGCAATACCACCACTAATCCGTGCCCCTATGCGGTAAAT  -22 +4          1  (1)
        CCGCATTAAAGTGGATGAGTGTGGTCGGCTGTGGGTTTTGGACA······                         ·········TCGGCAATACCACCACTAATCCGTGCCCCTATGCGGTAAAT  -15             1  (1)
        CCGCATTAAAGTGGATGAGTGTGGTCGGCTGTGGGTTTTGGACACTGG··                         ·············CAATACCACCACTAATCCGTGCCCCTATGCGGTAAAT  -15             1  (1)
        CCGCATTAAAGTGGATGAGTGTGGTCGGCTGTGGGTTTTGGACACTGG··                         ······GCATCGGCAATACCACCACTAATCCGTGCCCCTATGCGGTAAAT  -8              6  (3)
        CCGCATTAAAGTGGATGAGTGTGGTCGGCTGTGGGTTTTGGAC·······                         ·CGTGGGCATCGGCAATACCACCACTAATCCGTGCCCCTATGCGGTAAAT  -8              1  (1)
        CCGCATTAAAGTGGATGAGTGTGGTCGGCTGTGGGTTTTGGACAC·····                         ·CGTGGGCATCGGCAATACCACCACTAATCCGTGCCCCTATGCGGTAAAT  -6              3  (3)
        CCGCATTAAAGTGGATGAGTGTGGTCGGCTGTGGGTTTTGGACA······ T                       ·CGTGGGCATCGGCAATACCACCACTAATCCGTGCCCCTATGCGGTAAAT  -7  +1          1  (1)
        CCGCATTAAAGTGGATGAGTGTGGTCGGCTGTGGGTTTTGG········· GCAT                    CCGTGGGCATCGGCAATACCACCACTAATCCGTGCCCCTATGCGGTAAAT  -9  +4          1  (1)
        CCGCATTAAAGTGGATGAGTGTGGTCGGCTGTGGGTTTTGGACAC·····                         CCGTGGGCATCGGCAATACCACCACTAATCCGTGCCCCTATGCGGTAAAT  -5              1  (1)
        CCGCATTAAAGTGGATGAGTGTGGTCGGCTGTGGGTTTTGGACACTG···                         CCGTGGGCATCGGCAATACCACCACTAATCCGTGCCCCTATGCGGTAAAT  -3              3  (1)
        CCGCATTAAAGTGGATGAGTGTGGTCGGCTGTGGGTTTTGGACACTGGAA TGTC                    ·······CATCGGCAATACCACCACTAATCCGTGCCCCTATGCGGTAAAT  -7  +4          1  (1)
        CCGCATTAAAGTGGATGAGTGTGGTCGGCTGTGGGTTTTGGACACTGG··                         CCGTGGGCATCGGCAATACCACCACTAATCCGTGCCCCTATGCGGTAAAT  -2              1  (1)
   y1b: CCGCATTAAAGTGGATGAGTGTGGTCGGCTGTGGGTTTTGGACACTGGA·                         CCGTGGGCATCGGCAATACCACCACTAATCCGTGCCCCTATGCGGTAAAT  -1             18  (6)
        CCGCATTAAAGTGGATGAGTGTGGTCGGCTGTGGGTTTTGGACACTGG·· T                       CCGTGGGCATCGGCAATACCACCACTAATCCGTGCCCCTATGCGGTAAAT  -2  +1          1  (1)
        CCGCATTAAAGTGGATGAGTGTGGTCGGCTGTGGGTTTTGGACACTGGAA T                       ·CGTGGGCATCGGCAATACCACCACTAATCCGTGCCCCTATGCGGTAAAT  -1  +1   (y+)   2  (1)
        CCGCATTAAAGTGGATGAGTGTGGTCGGCTGTGGGTTTTGGACACTGGA· CAT                     ·CGTGGGCATCGGCAATACCACCACTAATCCGTGCCCCTATGCGGTAAAT  -2  +3          1  (1)
        CCGCATTAAAGTGGATGAGTGTGGTCGGCTGTGGGTTTTGGACACTGGA· CATTG                   CCGTGGGCATCGGCAATACCACCACTAATCCGTGCCCCTATGCGGTAAAT  -1  +5          1  (1)

DT-tGD(y1,w2,y1b,w2b) [pVMG129]
        CCGCATTAAAGTGGATGAGTGTGGTCGGCTGTGGGTTTTGGACACTGG··                         ·······CATCGGCAATACCACCACTAATCCGTGCCCCTATGCGGTAAAT  -9              1  (1)
        CCGCATTAAAGTGGATGAGTGTGGTCGGCTGTGGGTTTTGGACACTGG··                         ······GCATCGGCAATACCACCACTAATCCGTGCCCCTATGCGGTAAAT  -8              5  (2)
        CCGCATTAAAGTGGATGAGTGTGGTCGGCTGTGGGTTTTGGACAC·····                         CCGTGGGCATCGGCAATACCACCACTAATCCGTGCCCCTATGCGGTAAAT  -5              2  (1)
        CCGCATTAAAGTGGATGAGTGTGGTCGGCTGTGGGTTTTGGACACT····                         CCGTGGGCATCGGCAATACCACCACTAATCCGTGCCCCTATGCGGTAAAT  -4              1  (1)
        CCGCATTAAAGTGGATGAGTGTGGTCGGCTGTGGGTTTTGGACACTGG··                         ·CGTGGGCATCGGCAATACCACCACTAATCCGTGCCCCTATGCGGTAAAT  -3              1  (1)
        CCGCATTAAAGTGGATGAGTGTGGTCGGCTGTGGGTTTTGGACACTGGAA                         ··GTGGGCATCGGCAATACCACCACTAATCCGTGCCCCTATGCGGTAAAT  -2              7  (1)
        CCGCATTAAAGTGGATGAGTGTGGTCGGCTGTGGGTTTTGGACACTGGAA A                       CCGTGGGCATCGGCAATACCACCACTAATCCGTGCCCCTATGCGGTAAAT      +1          4  (2)
        CCGCATTAAAGTGGATGAGTGTGGTCGGCTGTGGGTTTTGGACACTGGA· CA                      CCGTGGGCATCGGCAATACCACCACTAATCCGTGCCCCTATGCGGTAAAT  -1  +2          2  (2)
        CCGCATTAAAGTGGATGAGTGTGGTCGGCTGTGGGTTTTGGACACTGGAA CA                      ·CGTGGGCATCGGCAATACCACCACTAATCCGTGCCCCTATGCGGTAAAT  -1  +2          2  (1)
        CCGCATTAAAGTGGATGAGTGTGGTCGGCTGTGGGTTTTGGACACTGGA· CACTTTTGGACA            CCGTGGGCATCGGCAATACCACCACTAATCCGTGCCCCTATGCGGTAAAT  -1  +12         2  (1)
```

Supplementary Figure 1 - Resistant allele sequences Resistant allele sequences recovered at the *white* locus **(a)** and *yellow* locus **(b)** with sections for each construct used. gRNAs present in each construct are in parentheses. Sequence complementary to the *w2*-gRNA **(a)** or the *y1*-gRNA **(b)** is in blue, PAM is in red, and sequence is split at the cut site. Wild-type sequence for comparison at the top of each panel. Dots represent missing bases; insertions are shown in green. The number of bases missing and/or inserted is noted to the right of each sequence. Flies that were *w+* or *y+* are marked as such. The number of flies and number of individual crosses from which each allele was recovered in each experiment are on the far right. The *w2b* allele is highlighted in pink and the *y1b* allele is highlighted in yellow.

Supplementary Figure 2

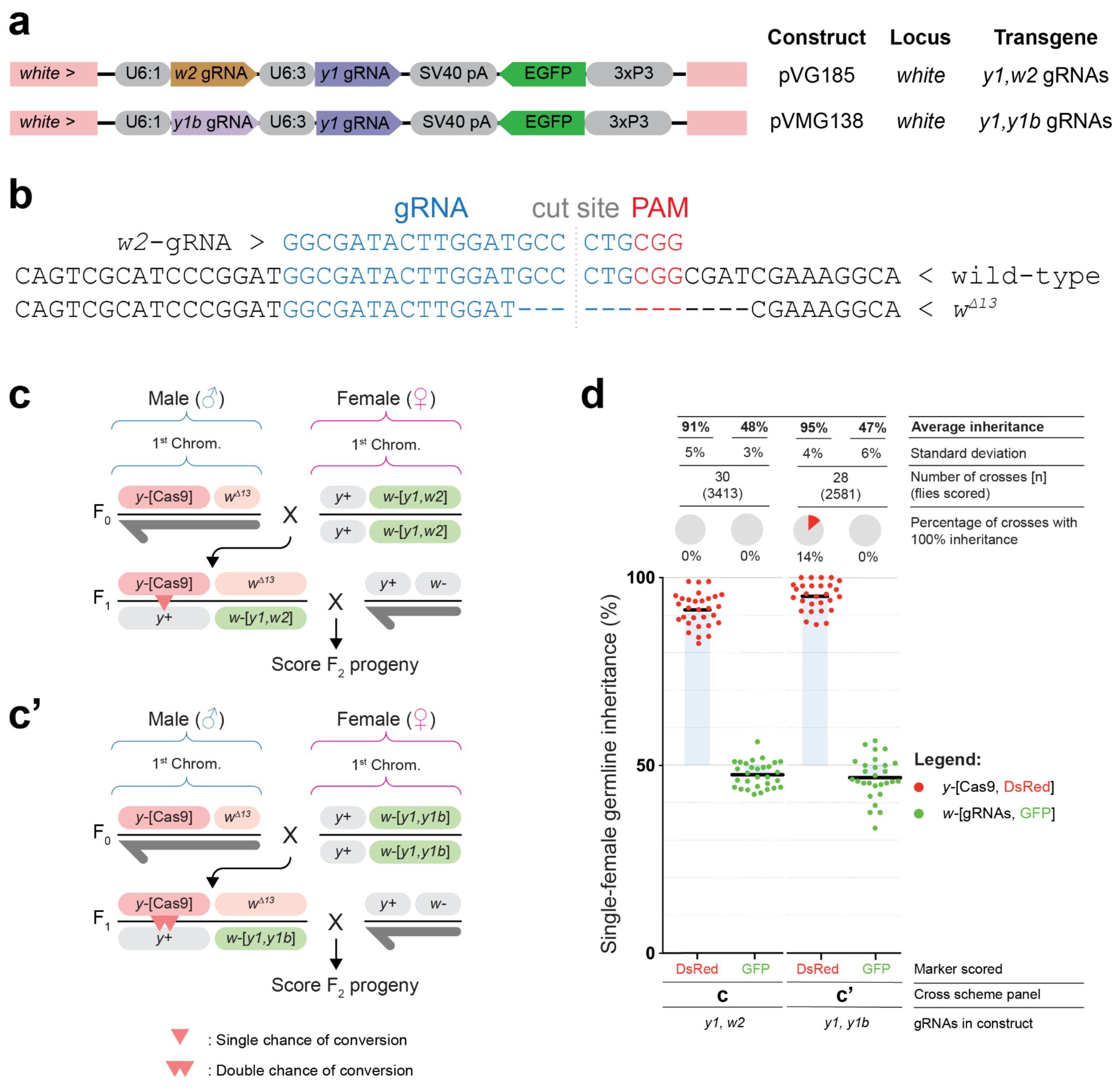

Supplementary Figure 2 - Testing double-tap in a condition in which the total number of gRNAs is held constant (a) Transgenic fly lines used in this experiment. Various gRNAs driven by U6 promoters and marked with 3xP3-EGFP are inserted at the *white* locus. **(b)** Sequence of the *w2*-gRNA aligned with *white* locus of the wild-type and $w^{\Delta13}$ strain. **(c and c')** Cross schemes used in this experiment. F_0 males carrying DsRed-marked Cas9 inserted at the *yellow* locus and the $w^{\Delta13}$ allele are crossed to virgin females carrying either the single-cutting **(c)** or double-tap **(c')** pair of GFP-marked gRNAs inserted at the *white* locus. Transheterozygous F_1 virgin females are single-pair crossed to wild-type males and the resulting progeny are scored for red and green fluorescence as markers of transgene inheritance. **(d)** Single female germline inheritance rates as measured by fluorescence markers in the F_2 flies. Graph labeled the same as **Figure 1e**.

Supplementary Figure 3

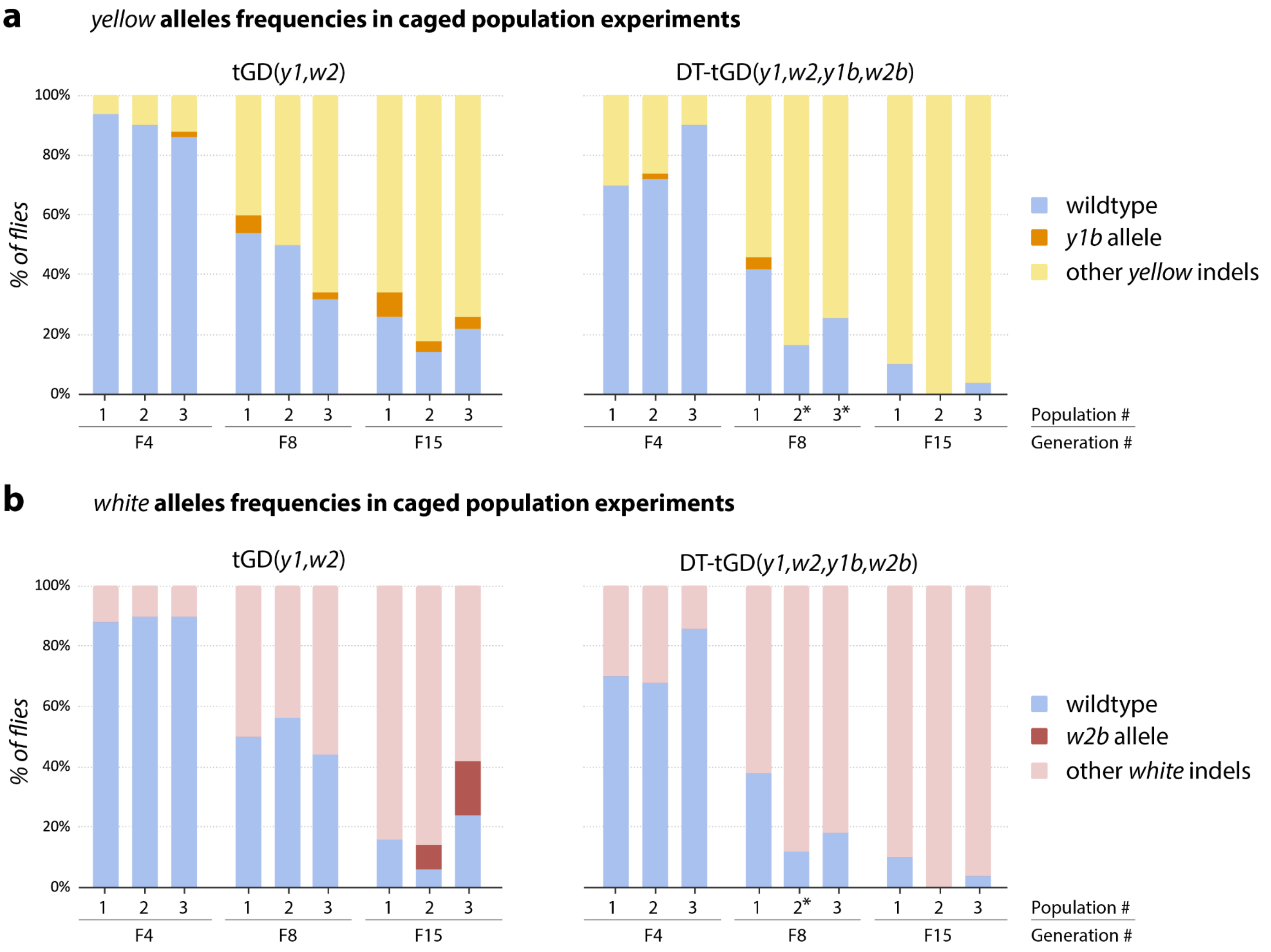

Supplementary Figure 2 - Analysis of indel generation during the spread of tGD and DT-tGD in caged populations. Analysis of indel allele generation at **(a)** the *yellow* and **(b)** *white* loci. In all conditions a genomic pool containing of 50 alleles (from 50 random males) was sampled, except for samples marked with an asterisk (*): 1) Population 2/F8, where 30 alleles were sampled for *yellow* and 42 for *white*; and 2) Population 3/F8 where 39 alleles were sampled for *yellow*.

SUPPLEMENTARY TABLES

Supplementary Table 1 - Primer List

Name	Primer
**Primers used for building constructs**	
373	TTTGCTCACCTGTGATTGCTCC
451	GTACGCGTATCGATAAGCTTTAAGATACATTGATGAGTTTGG
590	AAGCTGGAGCTCCTGCAGGTTGTTGGTTGGCACACC
V0181	CCTGCAGGAGCTCCAGCTTTTGTTAAAAAGAACAATATCGATATACGATATTTTAAGTTC
V0421	AACCGGTCCAGTGTCCAAAACCAAATGTAGCTCAAAAAATTGCGTATATAAATG
V0422	ACATTTGGTTTTGGACACTGGACCGGTTTTAGAGCTAGAAATAGCAAGTTAAAATAAGGC
V0423	GTATCTTAAAGCTTATCGATACGCGTACGCTAGCAATAAATGGCGACAGCAACAAAAAAC
V0424	CAGGCATCCAAGTATCGCCAAATGTAGCTCAAAAAATTGCGTATATAAATG
V0425	GGCGATACTTGGATGCCTGGTTTTAGAGCTAGAAATAGCAAGTTAAAATAAGGC
V0426	ACAACCTGCAGGAGCTCCAGCTTTTGTCGTACAACTAGGGTTGCAACCAGGTAGTTGGAC
V0433	CTAAAACCAGGCATCCAAGTATCGCCGAAGATTAGATCCTTAAATCTGCTATTTAAATGG
V0434	AACTTAAAATATCGTATATCGATATTGTTCTTTTTACAACAATTATAAAAAAAAGCGCAC
V0435	GTAAAAAGAACAATATCGATATACGATATTTTAAGTTCCTTTCGCGCTTAGCCATTACAC
V0442	GGAGCAATCACAGGTGAGCAAAAAAGACGTCTGCGGCGATCGAAAGG
V0489	GGAGCAATCACAGGTGAGCAAAAAAGCACCGACTCGG
V0490	CAGTATATATAGGAAAGATATCCGGGTGAACTTCGGTTTTGGACACTGGACCG
V0495	GAAGTTCACCCGGATATCTTTCCTATATATACTG
**Primers used for amplifying and sequencing individual *white* indels**	
V0478	GCTGGTCAACCGGACACGCGG
V0659	AGGGAGCCGATAAAGAGGTCATCC
**Primers used for amplifying and sequencing individual *yellow* indels**	
V0477	TATTATCCAGAAAACAGACAGC
V0657	GGACATACCAAATATACCCTCC
**Primers used for deep sequencing at *white* (adapter in green)**	
V1442	ACACTCTTTCCCTACACGACGCTCTTCCGATCTCTCTCTATTCGCAGTCGGCTGATCTG

V1519	TGGAGTTCAGACGTGTGCTCTTCCGATCTGGTCATCCTGCTGGACATAGGC
**Primers used for deep sequencing at *yellow* (adapter in green)**	
V1440	ACACTCTTTCCCTACACGACGCTCTTCCGATCTCTCTGCTAATTCCGTATCCAGATTGGC
V1518	TGGAGTTCAGACGTGTGCTCTTCCGATCTGCCTATATCCACGGCAATGTTAGC

SUPPLEMENTARY DATA

Supplementary Data 1 - DT-tGD with elements separate in F0

Raw counting data of the F2 progeny with phenotypic scoring for females, males, DsRed positive (DsRed), GFP positive (GFP), both fluorophores (both), or no fluorescence (none). The Cas9 transgene was tracked by DsRed presence and the gRNA transgene was tracked by GFP presence. Transgene inheritance rates in the F2 for each tube (marked as "F1 Cross" in table) were calculated by combining data from males and females. Average inheritance, standard deviation, and percentage of vials at 100% inheritance were calculated for each transgene as well. Any crosses that resulted in fewer than 30 progeny were excluded from these calculations and any further analysis. The data is subdivided into the following tabs:

1. **Fig. 1e - tGD(y1,w2):** Counting data and inheritance rates of the regular tGD.
2. **Fig. 1e - DT-tGD(y1,w2,y1b):** Counting data and inheritance rates of the DT-tGD with *y1b*.
3. **Fig. 1e - DT-tGD(y1,w2,w2b):** Counting data and inheritance rates of the DT-tGD with *w2b*.
4. **Fig. 1e - DT-tGD(y1,w2,y1b,w2b):** Counting data and inheritance rates of the DT-tGD with *y1b* and *w2b*.
5. **Fig. 1 - Summary:** Summary of the total flies and crosses counted and the average inheritance rates, standard deviations, and vials at 100% inheritance for each condition.
6. **Fig. 1 - Statistics (inheritance rates):** Kolmogorov-Smirnov tests for normal distribution and Mann Whitney tests for difference in means of inheritance rates of each transgene between the control tGD(y1,w2) and each double-tap condition.
7. **Fig. 1 - Statistics (pie charts):** Randomization tests for a difference in proportions of vials at 100% inheritance for each transgene between the control tGD(y1,w2) and each double-tap condition.

Supplementary Data 2 - DT-tGD with elements together in F0

Raw counting data of the F2 progeny with phenotypic scoring and analysis the same as Supplementary Data 1. The data is subdivided into the following tabs:

1. **Fig. 2c - tGD(y1,w2) a:** Counting data and inheritance rates of the regular tGD with both elements inherited paternally.
2. **Fig. 2c - DT-tGD(y1,w2,y1b,w2b) a:** Counting data and inheritance rates of the DT-tGD with both elements inherited paternally.
3. **Fig. 2c - tGD(y1,w2) b:** Counting data and inheritance rates of the regular tGD with both elements inherited maternally.
4. **Fig. 2c DT-tGD(y1,w2,y1b,w2b) b:** Counting data and inheritance rates of the DT-tGD with both elements inherited maternally.
5. **Fig. 2 - Summary:** Summary of the total flies and crosses counted and the average inheritance rates, standard deviations, and vials at 100% inheritance for each condition.
6. **Fig. 2 - Statistics (inheritance rates):** Kolmogorov-Smirnov tests for normal distribution and Mann Whitney tests for difference in means of inheritance rates of each transgene between the control paternal and double-tap paternal, and between the control maternal and double-tap maternal.
7. **Fig. 2 - Statistics (pie charts):** Randomization tests for a difference in proportions of vials at 100% inheritance of each transgene between the control paternal and double-tap paternal, and between the control maternal and double-tap maternal.

Supplementary Data 3 - C-tGD showing gRNAs cut as expected

Raw counting data of the F2 progeny with phenotypic scoring and analysis the same as Supplementary Data 1. The data is subdivided into the following tabs:

1. **Fig. 3d - C-tGD(y1b,w2):** Counting data and inheritance rates of the C-tGD when crossed to the Cas9 line.
2. **Fig. 3d - C-tGD(y1,w2b):** Counting data and inheritance rates of the C-tGD when crossed to the Cas9 line.
3. **Fig. 3e - C-tGD(y1b,w2):** Counting data and inheritance rates of the C-tGD when inherited together paternally with Cas9 and crossed to the *y1b*,*w2b* line.
4. **Fig. 3e - C-tGD(y1,w2b):** Counting data and inheritance rates of the C-tGD when inherited together paternally with Cas9 and crossed to the *y1b*,*w2b* line.
5. **Fig. 3 - Summary:** Summary of the total flies and crosses counted and the average inheritance rates, standard deviations, and vials at 100% inheritance for each condition.

Supplementary Data 4 - C-tGD holding number of gRNAs constant

Raw counting data of the F2 progeny with phenotypic scoring and analysis the same as Supplementary Data 1. The data is subdivided into the following tabs:

1. **Supp. Fig. 2d - tGD(y1,w2):** Counting data and inheritance rates of the regular tGD when crossed to the Cas9, $w^{\Delta 13}$ line.
2. **Supp. Fig. 2d - C-tGD(y1,y1b):** Counting data and inheritance rates of the C-tGD when crossed to the Cas9, $w^{\Delta 13}$ line.
3. **Supp. Fig. 2d - Summary:** Summary of the total flies and crosses counted and the average inheritance rates, standard deviations, and vials at 100% inheritance for each condition.
4. **Supp. Fig. 2 - Statistics (inheritance rates):** Kolmogorov-Smirnov tests for normal distribution and Mann Whitney tests for difference in means of inheritance rates of each transgene between the regular tGD(y1,w2) and C-tGD(y1,y1b).

Supplementary Data 5 - tGD and DT-tGD cages

Raw counting data of each generation from the cage experiments with phenotypic scoring for females, males, DsRed positive (DsRed), GFP positive (GFP), both fluorophores (both), or no fluorescence (none). The Cas9 transgene was tracked by DsRed presence and the gRNA transgene was tracked by GFP presence. Transgene inheritance rates were calculated by combining data from males and females. The data is subdivided into the following tabs:

1. **Fig. 4 - tGD(y1,w2) Bottles:** Counting data and inheritance rates of the regular tGD when spread in a population.
2. **Fig. 4 - DT-tGD(y1,w2,y1b,w2b) Bottles:** Counting data and inheritance rates of the DT-tGD when spread in a population.
3. **Fig. 4 - Comparison of bottles**: Summary and comparison of inheritance rates of each transgene in the tGD and DT-tGD bottles.

Supplementary Data 6 - *Yellow* deep sequencing indel analysis

Population cage deep sequencing results and analysis of indels at the *yellow* locus. The data is subdivided into the following tabs:

1. **Yellow Summary:** Summary of sequencing results including the prevalence of wildtype, *y1b*, and other alleles, and estimates of the number of flies with each allele.
2. **Yellow Oregon R Control:** Sequencing reads from wildtype control flies.
3. **tGD, Generation #, Bottle #:** Sequencing reads from the regular tGD cages, divided into tabs by generation and cage.

4. **DT-tGD, Generation #, Bottle #:** Sequencing reads from the DT-tGD cages, divided into tabs by generation and bottle.

Supplementary Data 7 - *White* deep sequencing indel analysis

Population cage deep sequencing results and analysis of indels at the *white* locus. The data is subdivided into the following tabs:

1. **White Summary:** Summary of sequencing results including the prevalence of wildtype, *w2b*, and other alleles, and estimates of the number of flies with each allele.
2. **White Oregon R Control:** Sequencing reads from wildtype control flies.
3. **tGD, Generation #, Bottle #:** Sequencing reads from the regular tGD cages, divided into tabs by generation and cage.
4. **DT-tGD, Generation #, Bottle #:** Sequencing reads from the DT-tGD cages, divided into tabs by generation and bottle.

SUPPLEMENTARY METHODS

Plasmid information and cloning details

1) **pVG182 *vasa*-Cas9 (Accession number: MN551085)**

   See López Del Amo et al., 2020, *Cell Reports*[1]

2) **pVG185 tGD(y1,w2) (Accession number: MN551090)**

   See López Del Amo et al., 2020, *Nature Communications*[2]

3) **pVMG127 DT-tGD(y1,w2,y1b) (Accession number: OL630771)**

   The entirety of pVG185, which contains *w2* homology arms (HAs), a 3xP3>GFP fluorescent marker, and *w2* and *y1* gRNAs driven by the *D. mel* U6-1 and U6-3 promoters, respectively, was amplified by PCR. The *D. gri* U6-C 5'UTR with y1b gRNA and the 3'UTR were each amplified by PCR from a *D. gri* U6-C plasmid. The three PCRs were combined by Gibson assembly.

   Primers used in this construct: 451, 590, V0421, V0181, V0422, V0423

4) **pVMG128 DT-tGD(y1,w2,w2b) (Accession number: OL630772)**

   The entirety of pVG185 was amplified by PCR. The *D. gri* U6-C 5'UTR with w2b gRNA and the 3'UTR were each amplified by PCR from a *D. gri* U6-C plasmid. The three PCRs were combined by Gibson assembly.

   Primers used in this construct: 451, 590, V0424, V0181, V0423, V0425

5) **pVMG129 DT-tGD(y1,w2,y1b,w2b) (Accession number: OL630773)**

   The entirety of pVMG127 was amplified by PCR. The *D. gri* U6-A 5'UTR with w2b gRNA and 3'UTR were each amplified by PCR from a D. gri U6-A plasmid. The three PCRs were combined by Gibson assembly.

   Primers used in this construct: 590, V0435, V0433, V0426, V0434, V0425

6) **pVMG130 C-tGD(w2,y1b) (Accession number: OL630774)**

   pVMG127 was amplified by PCR, excluding the *w2* and *y1* gRNAs and their promoters. *D. mel* U6-3 driving the *w2* gRNA was amplified from a pCFD3 plasmid containing *w2*. The two PCRs were combined by Gibson assembly.

   Primers used in this construct: V0181, V0442, 373, 590

7) **pVMG131 C-tGD(y1,w2b) (Accession number: OL630775)**

   pVMG128 was amplified by PCR, excluding the *w2* and *y1* gRNAs and their promoters. *D. mel* U6-3 driving the *y1* gRNA was amplified from a pCFD3 plasmid containing *y1*. The two PCRs were combined by Gibson assembly.

   Primers used in this construct: V0181, V0442, 373, 590

8) **pVMG138 C-tGD(y1,y1b) (Accession number: OL630776)**

   pVG185 was amplified by PCR, excluding the *w2* gRNA. The *y1b* gRNA was amplified by PCR from pVMG127. The two PCRs were combined by Gibson assembly.

   Primers used in this construct: 373, V0495, V0490, V0489

SUPPLEMENTARY REFERENCES

1. López Del Amo, V. *et al.* Small-Molecule Control of Super-Mendelian Inheritance in Gene Drives. *Cell Rep.* **31**, 107841 (2020).
2. López Del Amo, V. *et al.* A transcomplementing gene drive provides a flexible platform for laboratory investigation and potential field deployment. *Nat. Commun.* **11**, 352 (2020).
