# Supplementary material for: Double-tap gene drive uses iterative genome targeting to help overcome resistance alleles
Source: Nat Commun. 2022 May 9;13:2595. doi: 10.1038/s41467-022-29868-3 (PMC9085836; doi:10.1038/s41467-022-29868-3)
Supplement: Supplementary file 2 — Reporting Summary [file 41467_2022_29868_MOESM2_ESM.pdf]

nature portfolio

Corresponding author(s): Valentino M. Gantz

Last updated by author(s): Jan 23, 2022

Reporting Summary

Nature Portfolio wishes to improve the reproducibility of the work that we publish. This form provides structure for consistency and transparency in reporting. For further information on Nature Portfolio policies, see our Editorial Policies and the Editorial Policy Checklist.

Statistics

For all statistical analyses, confirm that the following items are present in the figure legend, table legend, main text, or Methods section.

n/a	Confirmed	
[ ]	[x]	The exact sample size (n) for each experimental group/condition, given as a discrete number and unit of measurement
[ ]	[x]	A statement on whether measurements were taken from distinct samples or whether the same sample was measured repeatedly
[ ]	[x]	The statistical test(s) used AND whether they are one- or two-sided<br>*Only common tests should be described solely by name; describe more complex techniques in the Methods section.*
[x]	[ ]	A description of all covariates tested
[x]	[ ]	A description of any assumptions or corrections, such as tests of normality and adjustment for multiple comparisons
[ ]	[x]	A full description of the statistical parameters including central tendency (e.g. means) or other basic estimates (e.g. regression coefficient) AND variation (e.g. standard deviation) or associated estimates of uncertainty (e.g. confidence intervals)
[ ]	[x]	For null hypothesis testing, the test statistic (e.g. F, t, r) with confidence intervals, effect sizes, degrees of freedom and P value noted<br>*Give P values as exact values whenever suitable.*
[x]	[ ]	For Bayesian analysis, information on the choice of priors and Markov chain Monte Carlo settings
[x]	[ ]	For hierarchical and complex designs, identification of the appropriate level for tests and full reporting of outcomes
[x]	[ ]	Estimates of effect sizes (e.g. Cohen's d, Pearson's r), indicating how they were calculated

Our web collection on statistics for biologists contains articles on many of the points above.

Software and code

Policy information about availability of computer code

Data collection	Microsoft Excel 2011, Microsoft Excel 16.16.6, and Google Sheets were used for data collection. Some of the collected data was analyzed using the CRISPResso online (Version 3) resource (https://crispresso.pinellolab.partners.org/)
Data analysis	Graphpad Prism (v9) and Adobe Illustrator Creative Cloud (v25.4.1) were used for data analysis and display. CRISPResso parameters used in our data analysis are provided in the Supplementary Data Sets. The additional software used for the statistical analysis was StatKey v. 2.1.1

For manuscripts utilizing custom algorithms or software that are central to the research but not yet described in published literature, software must be made available to editors and reviewers. We strongly encourage code deposition in a community repository (e.g. GitHub). See the Nature Portfolio guidelines for submitting code & software for further information.

Data

Policy information about availability of data

All manuscripts must include a data availability statement. This statement should provide the following information, where applicable:

- Accession codes, unique identifiers, or web links for publicly available datasets
- A description of any restrictions on data availability
- For clinical datasets or third party data, please ensure that the statement adheres to our policy

The plasmid sequences of the constructs generated in this manuscript are either deposited into the GenBank database. GenBank accession numbers for the deposited plasmids are the following: pVG182 vasa-Cas9 (MN551085)33, pVG185 tGD(y1,w2) (MN551090)19, pVMG127 DT-tGD(y1,w2,y1b) (OL630771), pVMG128 DT-tGD(y1,w2,w2b) (OL630772), pVMG129 DT-tGD(y1,w2,y1b,w2b) (OL630773), pVMG130 C-tGD(w2,y1b) (OL630774), pVMG131 C-tGD(y1,w2b) (OL630775), pVMG138 C-tGD(y1,y1b) (OL630776); additional information is provided in the Supplementary Information. All source data are provided along with this manuscript. They cover the raw phenotypical scoring data collected in the gene drive experiments, which are reported in the Supplementary Data 1-5 files, and the caged

population experiment deep-sequencing data in the Supplementary Data 6-7 files all in Microsoft Excel format (.xlsx). All other data and information is available upon request from the authors.

Field-specific reporting

Please select the one below that is the best fit for your research. If you are not sure, read the appropriate sections before making your selection.

- [x] Life sciences
- [ ] Behavioural & social sciences
- [ ] Ecological, evolutionary & environmental sciences

For a reference copy of the document with all sections, see nature.com/documents/nr-reporting-summary-flat.pdf

Life sciences study design

All studies must disclose on these points even when the disclosure is negative.

Sample size	For Gene drive experiments, in our previous experience of similar analysis of gene drive effect using single fly pair crosses a number size of >8 is usually representative, describing the overall behavior (Lopez del Amo et al. 2020, Nature Communications). As such for each of our experimental condition we have collected more than double the required data points (between 21-37 different samples).
Data exclusions	All raw data provided was included in the figures. Fruit fly crosses with no progeny due to contamination or other causes, were removed from the analysis and are not reported in the raw data tables. Crosses with less than 20 offspring were removed from the data plots in the images, although they are reported in the Supplementary Data files. The removal of crosses with low number of offspring was done to ensure that the inheritance value associated with each germline was representative of the whole potential offspring. The removed data points are present in the Supplementary Data files and are available to the readers for transparency.
Replication	All the drive inheritance experiments performed include 21-37 independent technical replicates plotted as individual data points.
Randomization	F1 female flies in separate experimental conditions, were randomly collected from different F0 crosses to perform each F1 cross.
Blinding	For all the experiments performed we have analyzed fluorescence presence in the eyes of fruit flies. This type of scoring does not need the investigators to be blind as the evaluation of the phenotype is presence or absence of the fluorescent marker and there is no much room for interpretation that could be subjective.

Reporting for specific materials, systems and methods

We require information from authors about some types of materials, experimental systems and methods used in many studies. Here, indicate whether each material, system or method listed is relevant to your study. If you are not sure if a list item applies to your research, read the appropriate section before selecting a response.

Materials & experimental systems

n/a	Involved in the study	
x		Antibodies
x		Eukaryotic cell lines
x		Palaeontology and archaeology
	x	Animals and other organisms
x		Human research participants
x		Clinical data
x		Dual use research of concern

Methods

n/a	Involved in the study	
x		ChIP-seq
x		Flow cytometry
x		MRI-based neuroimaging

Animals and other organisms

Policy information about studies involving animals; ARRIVE guidelines recommended for reporting animal research

Laboratory animals	All Drosophila melonogaster transgenic animals were generated or assembled in Oregon-R genetic background.
Wild animals	Study did not involve wild animals.
Field-collected samples	Study did not involve field-collected samples.
Ethics oversight	Drosophila melonogaster is an invertebrate and is exempt from IACUC oversight.

Note that full information on the approval of the study protocol must also be provided in the manuscript.
