# Supplementary material for: Double-tap gene drive uses iterative genome targeting to help overcome resistance alleles
Source: Nat Commun. 2022 May 9;13:2595. doi: 10.1038/s41467-022-29868-3 (PMC9085836; doi:10.1038/s41467-022-29868-3)
Supplement: Supplementary file 4 — Description of Additional Supplementary Files [file 41467_2022_29868_MOESM4_ESM.pdf]

Title: Supplementary Data 1 - DT-tGD with elements separate in F0

Description: Raw counting data of the F2 progeny with phenotypic scoring for females, males, DsRed positive (DsRed), GFP positive (GFP), both fluorophores (both), or no fluorescence (none). The Cas9 transgene was tracked by DsRed presence and the gRNA transgene was tracked by GFP presence. Transgene inheritance rates in the F2 for each tube (marked as "F1 Cross" in table) were calculated by combining data from males and females. Average inheritance, standard deviation, and percentage of vials at 100% inheritance were calculated for each transgene as well. Any crosses that resulted in fewer than 30 progeny were excluded from these calculations and any further analysis. The data is subdivided into the following tabs:

1. **Fig. 1e - tGD(y1,w2):** Counting data and inheritance rates of the regular tGD.
2. **Fig. 1e - DT-tGD(y1,w2,y1b):** Counting data and inheritance rates of the DT-tGD with *y1b*.
3. **Fig. 1e - DT-tGD(y1,w2,w2b):** Counting data and inheritance rates of the DT-tGD with *w2b*.
4. **Fig. 1e - DT-tGD(y1,w2,y1b,w2b):** Counting data and inheritance rates of the DT-tGD with *y1b* and *w2b*.
5. **Fig. 1 - Summary:** Summary of the total flies and crosses counted and the average inheritance rates, standard deviations, and vials at 100% inheritance for each condition.
6. **Fig. 1 - Statistics (inheritance rates):** Kolmogorov-Smirnov tests for normal distribution and Mann Whitney tests for difference in means of inheritance rates of each transgene between the control tGD(y1,w2) and each double-tap condition.
7. **Fig. 1 - Statistics (pie charts):** Randomization tests for a difference in proportions of vials at 100% inheritance for each transgene between the control tGD(y1,w2) and each double-tap condition.

Title: Supplementary Data 2 - DT-tGD with elements together in F0

Description: Raw counting data of the F2 progeny with phenotypic scoring and analysis the same as Supplementary Data 1. The data is subdivided into the following tabs:

1. **Fig. 2c - tGD(y1,w2) a:** Counting data and inheritance rates of the regular tGD with both elements inherited paternally.
2. **Fig. 2c - DT-tGD(y1,w2,y1b,w2b) a:** Counting data and inheritance rates of the DT-tGD with both elements inherited paternally.
3. **Fig. 2c - tGD(y1,w2) b:** Counting data and inheritance rates of the regular tGD with both elements inherited maternally.
4. **Fig. 2c DT-tGD(y1,w2,y1b,w2b) b:** Counting data and inheritance rates of the DT-tGD with both elements inherited maternally.
5. **Fig. 2 - Summary:** Summary of the total flies and crosses counted and the average inheritance rates, standard deviations, and vials at 100% inheritance for each condition.
6. **Fig. 2 - Statistics (inheritance rates):** Kolmogorov-Smirnov tests for normal distribution and Mann Whitney tests for difference in means of inheritance rates of each transgene between the control paternal and double-tap paternal, and between the control maternal and double-tap maternal.
7. **Fig. 2 - Statistics (pie charts):** Randomization tests for a difference in proportions of vials at 100% inheritance of each transgene between the control paternal and double-tap paternal, and between the control maternal and double-tap maternal.

Title: Supplementary Data 3 - C-tGD showing gRNAs cut as expected

Description: Raw counting data of the F2 progeny with phenotypic scoring and analysis the same as Supplementary Data 1. The data is subdivided into the following tabs:

1. **Fig. 3d - C-tGD(y1b,w2):** Counting data and inheritance rates of the C-tGD when crossed to the Cas9 line.
2. **Fig. 3d - C-tGD(y1,w2b):** Counting data and inheritance rates of the C-tGD when crossed to the Cas9 line.
3. **Fig. 3e - C-tGD(y1b,w2):** Counting data and inheritance rates of the C-tGD when inherited together paternally with Cas9 and crossed to the *y1b*,*w2b* line.
4. **Fig. 3e - C-tGD(y1,w2b):** Counting data and inheritance rates of the C-tGD when inherited together paternally with Cas9 and crossed to the *y1b*,*w2b* line.
5. **Fig. 3 - Summary:** Summary of the total flies and crosses counted and the average inheritance rates, standard deviations, and vials at 100% inheritance for each condition.

Title: Supplementary Data 4 - C-tGD holding number of gRNAs constant

Description: Raw counting data of the F2 progeny with phenotypic scoring and analysis the same as Supplementary Data 1. The data is subdivided into the following tabs:

1. **Supp. Fig. 2d - tGD(y1,w2):** Counting data and inheritance rates of the regular tGD when crossed to the Cas9, $w^{\Delta 13}$ line.
2. **Supp. Fig. 2d - C-tGD(y1,y1b):** Counting data and inheritance rates of the C-tGD when crossed to the Cas9, $w^{\Delta 13}$ line.
3. **Supp. Fig. 2d - Summary:** Summary of the total flies and crosses counted and the average inheritance rates, standard deviations, and vials at 100% inheritance for each condition.
4. **Supp. Fig. 2 - Statistics (inheritance rates):** Kolmogorov-Smirnov tests for normal distribution and Mann Whitney tests for difference in means of inheritance rates of each transgene between the regular tGD(y1,w2) and C-tGD(y1,y1b).

Title: Supplementary Data 5 - tGD and DT-tGD cages

Description: Raw counting data of each generation from the cage experiments with phenotypic scoring for females, males, DsRed positive (DsRed), GFP positive (GFP), both fluorophores (both), or no fluorescence (none). The Cas9 transgene was tracked by DsRed presence and the gRNA transgene was tracked by GFP presence. Transgene inheritance rates were calculated by combining data from males and females. The data is subdivided into the following tabs:

1. **Fig. 4 - tGD(y1,w2) Bottles:** Counting data and inheritance rates of the regular tGD when spread in a population.
2. **Fig. 4 - DT-tGD(y1,w2,y1b,w2b) Bottles:** Counting data and inheritance rates of the DT-tGD when spread in a population.
3. **Fig. 4 - Comparison of bottles**: Summary and comparison of inheritance rates of each transgene in the tGD and DT-tGD bottles.

Title: Supplementary Data 6 - *Yellow* deep sequencing indel analysis

Description: Population cage deep sequencing results and analysis of indels at the *yellow* locus. The data is subdivided into the following tabs:

1. **Yellow Summary:** Summary of sequencing results including the prevalence of wildtype, *y1b*, and other alleles, and estimates of the number of flies with each allele.
2. **Yellow Oregon R Control:** Sequencing reads from wildtype control flies.
3. **tGD, Generation #, Bottle #:** Sequencing reads from the regular tGD cages, divided into tabs by generation and cage.

4. **DT-tGD, Generation #, Bottle #:** Sequencing reads from the DT-tGD cages, divided into tabs by generation and bottle.

Title: Supplementary Data 7 - *White* deep sequencing indel analysis

Description: Population cage deep sequencing results and analysis of indels at the *white* locus. The data is subdivided into the following tabs:

1. **White Summary:** Summary of sequencing results including the prevalence of wildtype, *w2b*, and other alleles, and estimates of the number of flies with each allele.
2. **White Oregon R Control:** Sequencing reads from wildtype control flies.
3. **tGD, Generation #, Bottle #:** Sequencing reads from the regular tGD cages, divided into tabs by generation and cage.
4. **DT-tGD, Generation #, Bottle #:** Sequencing reads from the DT-tGD cages, divided into tabs by generation and bottle.
